# Robotic-assisted pedicle screw placement achieves high accuracy and narrows the experience gap: a preclinical evaluation

**DOI:** 10.1007/s11701-026-03513-3

**Published:** 2026-06-01

**Authors:** Anna T. Reza, Matthias Walper, Mitchell K. Ng, Paul G. Mastrokostas, Andrew S. Lee, Rafael G. de Oliveira, Kornelis A. Poelstra, John K. Ratliff, Rajiv K. Sethi, Michael A. Mont

**Affiliations:** 1 Stryker (United States), Kalamazoo, USA; 2 Rothman Orthopaedic Institute, Thomas Jefferson University Hospital, Philadelphia, USA; 3 Department of Orthopaedic Surgery, Maimonides Medical Center, Brooklyn, USA; 4 The Robotic Spine Institute of Las Vegas, Allegiant Spine Institute, Las Vegas, USA; 5 Stanford University, Stanford, USA; 6 Virginia Mason Medical Center, Seattle, USA; 7 Department of Orthopaedic Surgery, Rubin Institute for Advanced Orthopedics, Baltimore, USA

**Keywords:** Robot-assisted spine surgery, Pedicle screw placement, Accuracy, Gertzbein-Robbins classification, Surgical training

## Abstract

To assess a robotic-assisted system versus open manual techniques to: (1) compare pedicle screw accuracy between fellows and attendings with and without robotic assistance, and (2) evaluate executed versus planned trajectory fidelity relative to prior reports. Robotic-assisted spine systems aim to improve the accuracy, reproducibility, and safety of pedicle screw placement. Limited evidence compares outcomes across differing surgeon experience levels. A total of 255 thoracolumbar pedicle screws were placed in synthetic torsos simulating adult degenerative anatomy. Four surgeons (two fellows, two attendings) each placed half their screws using open fluoroscopy-guided techniques and half using a minimally invasive percutaneous approach with the Mako Spine system and intraoperative CT-based planning. Postoperative CT and the Gertzbein-Robbins classification were used to assess accuracy. Optimal placement (Grade A), breach rates, and positional/angular deviation from planned trajectories were recorded. The robotic cohort achieved a higher rate of clinically acceptable screw placement compared with the manual cohort, although this difference was not statistically significant (124/127, 97.6% vs. 120/128, 93.8%; *P* = 0.223). Fellows and attendings both achieved high clinically acceptable screw placement rates using robotics (63/64, 98.4% vs. 61/63, 96.8%). Among fellows, robotic assistance increased optimal screw placement compared with the manual technique (53/64, 82.8% vs. 41/64, 64.1%; *P* < 0.05). Robotic screws demonstrated low mean deviations from planned trajectories (1.4 ± 0.9 mm positional, 1.7 ± 1.1° angular). The Mako Spine system enabled precise screw placement and narrowed performance gaps for less experienced surgeons. It shows promise as both training tool and means to standardize outcomes.

## Introduction

Pedicle screw fixation remains fundamental to spinal instrumentation, yet achieving precise screw placement remains technically demanding, especially in patients who have challenging anatomy, including severe spondylosis or congenitally narrow pedicles [1]. Malpositioned screws may compromise biomechanical construct stability and increase the risk of neurologic injury, necessitating costly revisions [2, 3]. While traditional freehand and/or fluoroscopic guidance remains widely used, its limitations, including variable accuracy and increased radiation exposure, have prompted a growing adoption of navigation and robotic technologies [4–8]. 

Robotic-assisted platforms offer the potential for highly accurate and reproducible screw placement by integrating preoperative planning with intraoperative execution [7, 9, 10]. Early clinical and preclinical studies have demonstrated improvements in screw accuracy, reductions in cortical breach rates, and enhanced workflow consistency with robotic systems compared to conventional techniques [9, 11–13]. In a preclinical study of ten spine surgeons placing 160 pedicle screws total, Vaccaro et al. reported that robotic assistance was associated with improved accuracy (zero breaches versus 13, *P* < 0.05) and decreased radiation exposure (zero fluoroscopy images versus 108) in both open and minimally invasive cases [5]. Additional studies have suggested that robotic guidance may support longer and more medialized screws, optimize construct stability, and decrease radiation exposure to the surgical team, with added benefit in minimally invasive exposures [14–17]. A single institution study of 151 patients undergoing thoraco-lumbar pedicle screw fixation found that screws in the robotic group were longer (47.8 ± 6.4 versus 45.7 ± 4.3 mm; *P* < 0.001), wider (7 ± 0.7 versus 6.5 ± 0.3 mm; *P* < 0.001), and had higher stimulation thresholds (34.0 ± 11.9 versus 30.2 ± 9.8 mA; *P =* 0.002), indicating a greater safety margin from neural elements [14]. 

Much of the existing literature has focused on experienced surgeons performing robotic procedures in clinical settings [3, 18–21]. There remains limited evidence on how robotic systems perform in a controlled preclinical environment [5] or whether they can meaningfully narrow the performance gap between novice and experienced spine surgeons [7]. The potential for robotic platforms to elevate accuracy in less experienced users, particularly within the context of degenerative spine anatomy, has not been systematically examined.

To address these questions, this preclinical study assessed pedicle screw placement using the Mako Spine robotic-assisted platform in standardized synthetic models representing adult degenerative spine anatomy. Specifically, our aims were to: (1a) compare overall pedicle screw placement accuracy, between fellows and attendings with/without robotic assistance, (1b) relative to previous published studies; and to (2a) evaluate the impact of surgeon experience (attending versus fellow) on accuracy within each approach and (2b) relative to previous published studies.

## Methods

### Study cohorts

This study was a preclinical investigation performed using synthetic spine models to evaluate the accuracy of robotic-assisted versus freehand pedicle screw placement. As the study did not involve human or live animal subjects, formal Institutional Review Board approval was not required. However, the protocol underwent internal review and approval by Stryker’s research compliance committee (Stryker, Inc., Kalamazoo, Michigan). The experimental setup utilized 16 synthetic torsos (SurgiSTUD, Adult Degenerative Spine model, SurgiSTUD, Tempe, Arizona) designed to replicate consistent, reproducible adult spinal deformity anatomy from Thoracic 10 to Lumbar 5 (T10 to L5). There were four spine surgeons who participated: two senior attending surgeons (KAP, RKS) with over 10 years of post-certification experience and two early-career surgeons deemed “fellows” - one neurosurgery fellow (RGO) and one junior attending less than one year into practice (ASL). Each surgeon placed 32 pedicle screws (Everest, VB Spine) via a standard open, fluoroscopy-guided manual approach and 32 percutaneous pedicle screws (Everest XT, VB Spine) using a minimally-invasive approach with the Mako Spine system with Spine Guidance Software (version 5.2–21/021, Stryker Inc., Kalamazoo, Michigan) and the Mako 4 robotic arm (Stryker Inc., Kalamazoo, Michigan) with computed tomography (CT) (Airo TruCT, Stryker) imaging. Screw placement was performed according to a standardized protocol, with each surgeon placing screws using both techniques to reduce between-surgeon variability. All procedures were performed in a controlled laboratory environment. The use of standardized synthetic degenerative spine models allowed each surgeon to perform screw placement under reproducible anatomic conditions, thereby reducing variability related to patient-specific anatomy, bone quality, and intraoperative exposure. However, the manual and robotic cohorts differed by surgical approach: manual screws were placed using an open fluoroscopy-guided technique, whereas robotic-assisted screws were placed using a minimally invasive percutaneous technique. Therefore, comparisons should be interpreted as comparisons of the tested workflows rather than isolated comparisons of robotic guidance alone. Surgeon inclusion criteria included clinical experience of 10-plus years after board certification, while fellows had no prior experience with the Mako Spine system to preserve a naïve user baseline. The two fellows had no prior experience with the Mako Spine system, which was intended to preserve a naïve user baseline and allow evaluation of whether robotic assistance could support less experienced users in a standardized preclinical setting.

### Outcome measures

The principal outcome evaluated in this study was pedicle screw placement accuracy, determined through postoperative CT imaging and assessed by an independent spine neurosurgeon blinded to both surgical technique and surgeon experience level. Accuracy was assessed using the Gertzbein-Robbins classification system [22], which grades screws based on the degree of cortical breach [23]. Grade A indicates the screw is fully contained within the pedicle, Grade B reflects a breach of ≤ two mm, Grade C corresponds to a breach > two mm and ≤ four mm, Grade D represents a breach > four mm and ≤ six mm, and Grade E involves a breach > six mm. For this study, screws graded as A or B were defined as clinically acceptable, consistent with thresholds used in prior surgical accuracy literature, while Grade A screws were further categorized as optimal placements [23, 24]. A literature search was conducted to identify studies from January 2018 to June 2025 using combinations of the keywords “robotic pedicle screw,” “pedicle screw accuracy,” and “trajectory deviation” to identify peer-reviewed studies reporting rates of clinically acceptable screw placement and planned-versus-executed trajectory metrics. Relevant studies were reviewed and summarized for inclusion.

**Fig. 1:**
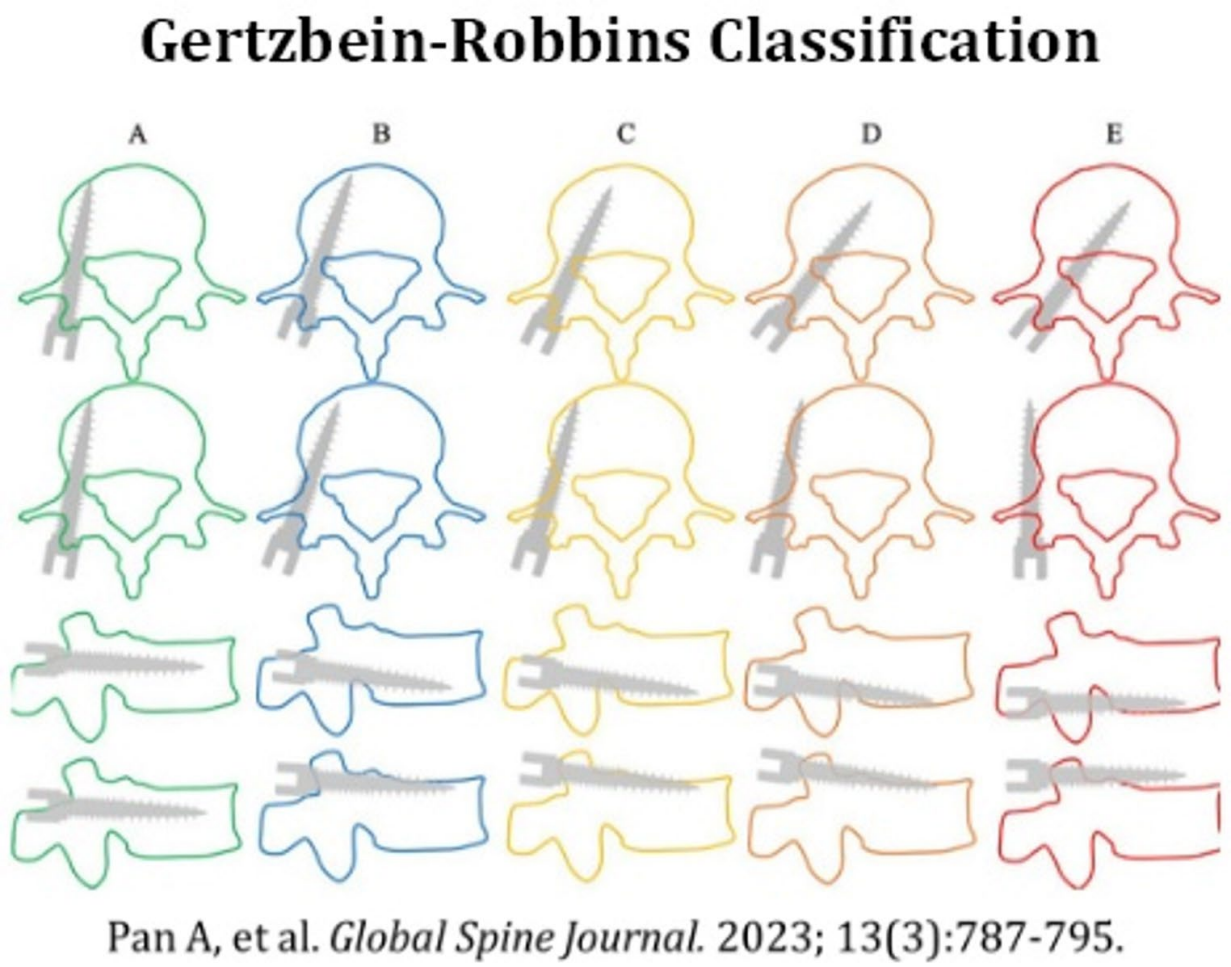
Gertzbein-Robbins Classification [22]

The secondary analyses focused on several dimensions of technical performance and workflow efficiency. These included the proportion of perfectly placed screws (Grade A only), as well as the fidelity of robotic execution to the preoperatively planned screw trajectories. For the robotic cohort, deviations between planned and executed trajectories were quantified by calculating both the linear displacement (in millimeters) and angular deviation (in degrees) of each screw (Fig. 1). The linear displacement was measured in the pedicle, which is assumed to be 20 mm beneath the executed screw head.

### Data analyses

Continuous variables, including positional deviation and angular deviation, were summarized using means and standard deviations. Categorical variables, including rates of clinically acceptable screw placement, defined as Gertzbein-Robbins Grades A and B, optimal placement, defined as Grade A only, and breaches greater than two mm, were reported as counts and percentages. Screw-level comparisons between robotic-assisted and manual techniques were performed using generalized linear models with a binomial distribution and logit link, with robust standard errors to account for clustering of screws within surgeon. Surgical technique, surgeon experience level, and their interaction were evaluated as predictors of screw accuracy. Given the limited number of participating surgeons, experience-stratified analyses were considered exploratory. Effect estimates were reported as absolute differences with 95% confidence intervals where appropriate, and statistical significance was set at *P* < 0.05. For the robotic group, deviation from the planned screw trajectory was analyzed descriptively by calculating the mean linear offset in millimeters and angular deviation in degrees between preoperative plans and postoperative CT-confirmed execution. All statistical analyses were performed using standard statistical software. All statistical analyses were conducted using standard software (e.g., Statistical Package for the Social Sciences (SPSS) or R).

## Results

### Overall pedicle screw accuracy: robotic versus manual

A total of 255 pedicle screws were evaluated; 128 placed via the open manual technique and 127 using the minimally invasive percutaneous technique with the Mako Spine robotic-assisted system. One robotic screw was excluded after intraoperative removal at the surgeon’s discretion because it was not retained as a final implanted screw and therefore did not have a finalized postoperative trajectory suitable for Gertzbein–Robbins grading or planned-versus-executed trajectory analysis. Overall, robotic screw placement demonstrated a higher rate of clinically acceptable placement compared with manual placement, although this difference was not statistically significant (124/127, 97.6% vs. 120/128, 93.8%; absolute difference, 3.9% points; 95% CI, -1.2 to 9.0; *P* = 0.223) (Table 1). Although this difference was not statistically significant, the robotic technique trended toward greater accuracy. Based on subgroup counts, the overall rate of optimal screw placement was similar between robotic and manual techniques (93/127, 73.2% vs. 91/128, 71.1%; absolute difference, 2.1% points; 95% CI, -8.7 to 12.9), although the effect differed by surgeon experience level.

**Table 1:** Overall Accuracy Results

	Overall *n*/*N* (%)	Surgeon Experience *n*/*N* (%)	
		Attending	Fellow
Open Manual	120/128 (93.8%)	59/64 (92.2%)	61/64 (95.3%)
MI Robotic-assisted	124/127 (97.6%)	61/63 (96.8%)	63/64 (98.4%)

MI: minimally invasive

### Accuracy by surgeon experience level

When stratified by surgeon experience, both fellows and attendings achieved similarly high rates of clinically acceptable screw placement using robotics (fellows: 63/64, 98.4%; attendings: 61/63, 96.8%) (Table 2).

**Table 2:** Impact of Surgeon Experience Level within Surgical Technique

Accuracy Metric	MI Robotic-Assisted–Attending *n*/*N* (%)	MI Robotic-Assisted–Fellow *n*/*N* (%)	Open Manual–Attending *n*/*N* (%)	Open Manual – Fellow *n*/*N* (%)
Clinically Acceptable (≤ 2 mm breach)	61/63 (96.8%)	63/64 (98.4%)	59/64 (92.2%)	61/64 (95.3%)
Optimal (no breach)	40/63 (63.5%)	53/64 (82.8%)	50/64 (78.1%)	41/64 (64.1%)
> 2 mm breach / Gertzbein–Robbins Grade C–E	2/63 (3.2%)	1/64 (1.6%)	5/64 (7.8%)	3/64 (4.7%)

The > 2 mm breach category corresponds to Gertzbein–Robbins Grades C, D, and E; grade-specific counts within this category were not available

MI: minimally invasive

### Attending improvement with robotic assistance

Among attending surgeons, robotic assistance increased clinically acceptable screw placement from 59/64 (92.2%) with the open manual technique to 61/63 (96.8%) with the minimally invasive robotic-assisted technique, although this comparison was not statistically significant (Table 3). In addition, the rate of significant cortical breaches (> two mm) decreased from 5/64 (7.8%) with manual placement to 2/63 (3.2%) with robotic assistance. To this end, our findings suggest that even experienced surgeons may benefit from robotic support in terms of overall safety and precision.

**Table 3:** Impact of Surgical Technique by Surgeon Experience Level

Accuracy Metric	Attending – Open Manual *n*/*N* (%)	Attending – MI Robotic *n*/*N* (%)	Fellow – Open Manual *n*/*N* (%)	Fellow – MI Robotic *n*/*N* (%)
Clinically Acceptable (≤ 2 mm breach)	59/64 (92.2%)	61/63 (96.8%)	61/64 (95.3%)	63/64 (98.4%)
Optimal (no breach)	50/64 (78.1%)	40/63 (63.5%)	41/64 (64.1%)	53/64 (82.8%)
> 2 mm breach / Gertzbein–Robbins Grade C–E	5/64 (7.8%)	2/63 (3.2%)	3/64 (4.7%)	1/64 (1.6%)

The > 2 mm breach category corresponds to Gertzbein–Robbins Grades C, D, and E; grade-specific counts within this category were not available

MI: minimally invasive

### Fellow improvement with robotic assistance

When analyzed from the reverse perspective—comparing techniques within each surgeon type—the benefit of robotics was clearest among fellows. They demonstrated an approximately 19% absolute increase in optimal (Grade A) screw placement using robotic assistance compared with manual placement (53/64, 82.8% vs. 41/64, 64.1%; *P* < 0.05) (Table 3), while also increasing clinically acceptable placement (63/64, 98.4% vs. 61/64, 95.3%) and reducing the rate of > two mm cortical breaches from 3/64 (4.7%) to 1/64 (1.6%). Taken together, our findings suggest that robotic guidance may reduce variability in screw placement and improve performance among less experienced surgeons.

These results are consistent with prior literature (Table 4), which has accuracy rates as high as 96 to 98% [11].

**Table 4:** Accuracy Relative to Other Robotic Studies since 2019

Authors (year)	Year	Number Screws	Outcomes
Vaccaro et al. [5]	2019	160	MIS: Robotic versus Manual (97.5 versus 80.0%) Open: Robotic versus Manual (92.5 versus 67.5%)
Huntsman et al. [6]	2019	562	Navigated robotic guidance achieved a successful pedicle screw placement rate of 98.8%
Vardiman et al. [7]	2020	630	Overall accuracy of screws 98.67% for left and 97.6% for right pedicle screws
Vardiman et al. [17]	2020	348	Overall, 97.4% (339 of 348) successful screw placement rate, 97.7% grade A/B clinically accuracy
Maalouly et al. [15]	2021	250	Overall, 98% accuracy (226 grade A/B, 24 grade C)

### Trajectory deviation (robotic group only)

**Fig. 2:**
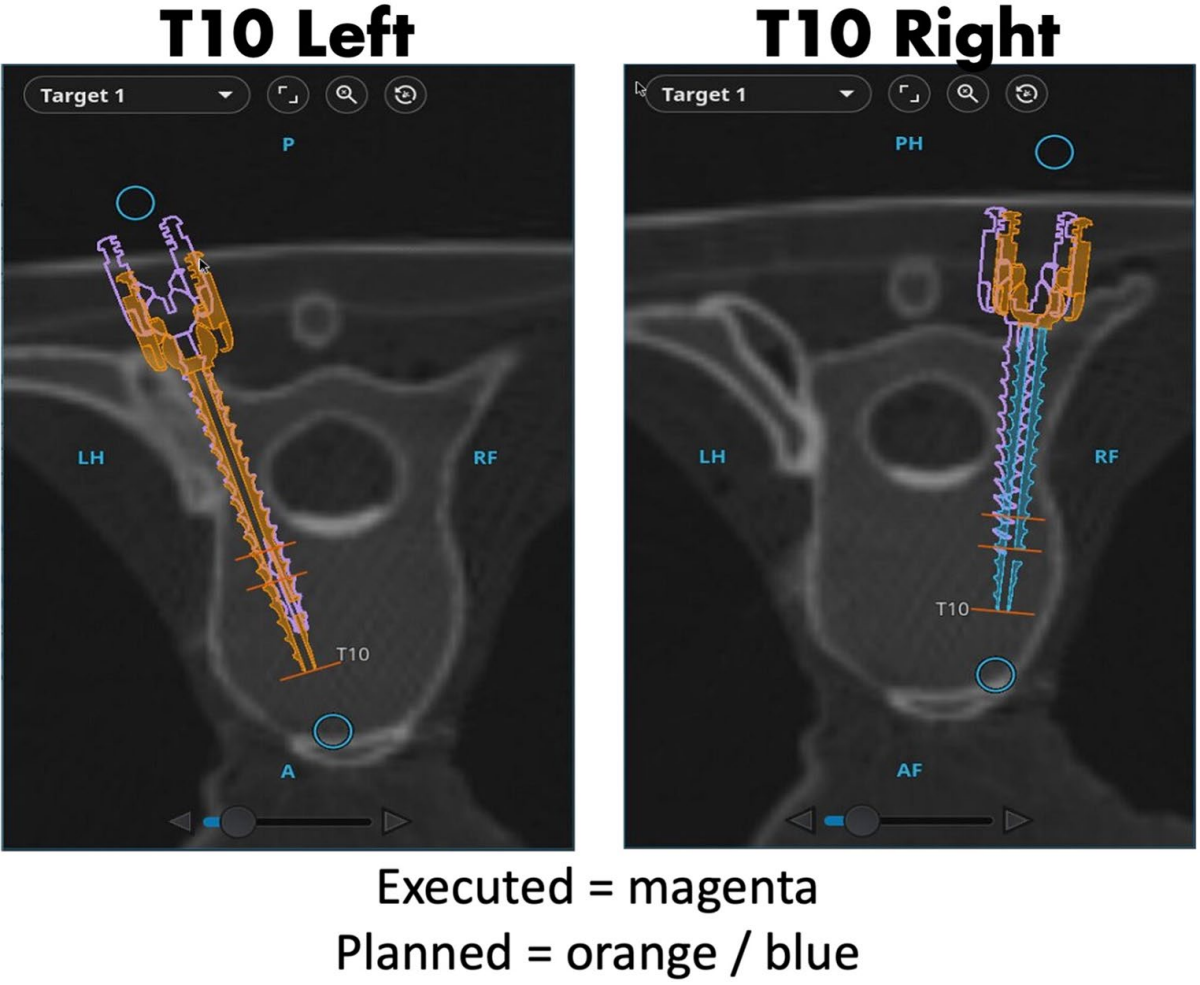
Example of Planned versus Executed Trajectories in Robotic Group

Analysis of planned versus executed trajectories in the robotic group revealed high fidelity to the surgical plan (Fig. 2). The mean positional deviation was 1.4 ± 0.9 mm, and the mean angular deviation was 1.7 ± 1.1°. Nearly half (47%) of screws deviated ≤ one mm from the planned path, and 59% deviated ≤ 1.5° in trajectory. These findings were consistent across all surgeons and spinal levels, indicating reproducible execution independent of surgeon experience.

Overall, our trajectory deviation results are equivalent if not superior to prior literature (Table 5), which generally reports positional deviation approximately two mm in each plane and two degrees of angulation.

**Table 5:** Trajectory outcomes of prior robotic studies

Authors	Year	Number Screws	Outcomes
Jiang et al. [13]	2018	8	Mean screw tip deviation 2.1 mm, mean screw head deviation 3.2 mm, mean angular offset 2.4°
Vaccaro et al. [5]	2019	160	MIS tip of screw positional deviation 1.58 ± 1.64 anterior-posterior and 1.63 ± 1.10 cranial-caudal. Angular difference in planned versus executed robotic-assisted screws were 2.39 ± 2.95° for MIS and 1.03 ± 1.09° for open.
Benech et al. [8]	2020	292	Average offset from preoperative plan to final screw placement was 1.9 ± 1.6 mm from screw tip, 2.3 ± 1.6 mm from screw head, and 2.8 ± 2.3° of angulation.
Wallace et al. [18]	2020	630	Average offset from preoperative plan 1.7 ± 1.3 mm from screw tip, 1.8 ± 1.2 mm from screw head, 2.0 ± 1.6 degrees of angulation
Benech et al. [21]	2022	726	Average offset from preoperative plan to final screw placement was 1.9 ± 1.5 mm from screw tip, 2.2 ± 1.4 mm from screw head and 2.9 ± 2.3° of angulation.
Vardiman et al. [19]	2023	75	Planned versus Executed deviation: 1.8 ± 1.3 mm for screw tip, 1.6 ± 0.9 mm for screw head, 2.1 ± 1.5 mm degrees of angulation

## Discussion

This preclinical study evaluated the accuracy and reproducibility of robotic-assisted pedicle screw placement using the Mako Spine system for percutaneous pedicle screw placement compared to conventional open manual techniques in synthetic models simulating adult degenerative spine anatomy. A total of 255 screws were analyzed, with fellows and attendings placing an equal number of screws using both methods. Overall, the robotic cohort achieved a higher rate of clinically acceptable screws (97.6%) compared to the manual cohort (93.8%), although this difference was not significant. Of note, the rate of optimal (Grade A) screws was significantly higher among fellows when using the robotic system (82.8% versus 64.1%, *P* < 0.05), indicating a performance enhancement in less experienced users, despite the technical challenges of minimally invasive techniques. This improvement in screw placement accuracy with robotic assistance occurred, despite the technical challenges of minimally invasive techniques, in comparison to the gross visualization afforded by open manual methods. In contrast, attendings achieved a higher optimal rate with the manual technique (78 versus 64%), though robotics improved their clinically acceptable screw rate (from 92 to 97%) and reduced cortical breach frequency. Across both cohorts, trajectory fidelity in the robotic group was high, with mean positional and angular deviations of 1.4 mm and 1.7°, respectively. Interestingly, fellows demonstrated numerically higher rates of clinically acceptable and optimal screw placement than attendings in the minimally invasive robotic-assisted cohort, whereas attendings achieved a higher rate of optimal placement in the open manual cohort. These differences were not statistically significant and should be interpreted cautiously given the small number of participating surgeons and the controlled synthetic model environment. One possible explanation is that attending surgeons may have derived greater relative benefit from direct visualization and prior experience with open manual techniques, whereas fellows may have benefited more from the structured planning, constrained trajectory guidance, and standardized workflow provided by robotic assistance. However, because surgeon-level sample size was limited, these findings should be viewed as exploratory and hypothesis-generating rather than evidence of superior performance by either experience group.

Our study aimed to compare the overall accuracy of pedicle screw placement between minimally invasive robotic-assisted and open manual techniques. While the robotic technique trended toward higher accuracy (97.6 versus 93.8%), this difference was not statistically significant, suggesting that experienced surgeons may be capable of achieving high levels of accuracy even with traditional methods. These results are in accordance with accuracy rates reported in prior robotic studies, which have demonstrated overall screw accuracy in the 96 to 98% range^5^ A retrospective study of 41 robotic-assisted spinal fusion surgeries found an overall high degree of accuracy (95%) of pedicle screw placement with a learning curve around 25 cases [15]. Of note, a key distinction in our current study is the stratified analysis by surgeon experience, which revealed that Mako Spine significantly improved accuracy in early-career surgeons—an outcome not consistently emphasized in earlier literature [19]. A retrospective review of 106 robotic-assisted spine surgeries with 630 pedicle screws found both resident and attending surgeons placed pedicle screws successfully under navigated robotic guidance (98.7% accuracy for attending versus 97.6% for resident) [19] Our data suggests that the Mako Spine may offer meaningful training value by narrowing the experience gap and standardizing outcomes across users. Such performance leveling is crucial in teaching hospitals and community settings where variability can impact safety and quality.

Our second aim was to assess whether robotic assistance could provide consistent trajectory execution aligned with preoperative plans. Analysis of the robotic cohort showed strong trajectory fidelity, with a mean positional deviation of 1.4 mm and angular deviation of 1.7°. These values are consistent with—and in some cases better than—trajectory deviation data from previously reported platforms, which typically cite deviations under two mm and 2°.^17^ There was one study of eight screws placed in L4-5 fusion cases that found a mean screw tip deviation of 2.1 mm, a mean screw head deviation of 3.2 mm, and a mean angular offset of 2.4 degrees [13]. In this study, 47% of screws deviated ≤ one mm from the planned pathway, and 73% deviated ≤ 2°, indicating precise alignment even in synthetic spines with degenerative deformity. These findings suggest that, within this controlled preclinical model, the Mako Spine system was able to preserve surgeon intent and reproduce planned trajectories with a high degree of consistency. Published preclinical claims from other platforms have suggested benefits such as “enhanced visualization” and “improved medialization and screw length,” but those gains are only clinically relevant if execution precision is maintained [5, 16].

Mako’s ability to deliver plan fidelity consistently across surgeon experience levels, while keeping trajectory deviations well within clinically safe thresholds, suggests a distinct advantage in both MIS workflows and other anatomically constrained regions. In our study, the manual group used an open approach with direct visualization, while the robotic group was performed minimally invasively. Prior reports have suggested that manual MI screw placement can be less accurate than open techniques because of limited exposure and reliance on fluoroscopy. However, MIS approaches offer important clinical advantages, including reduced blood loss, less soft-tissue disruption, and faster recovery. The fact that robotic-assisted MIS placement achieved accuracy comparable to or exceeding open manual techniques highlights how robotics can preserve these benefits without compromising precision or safety.

Several potential limitations should be considered when interpreting the results of this study. First, the use of synthetic spine models—while allowing for controlled comparisons—does not replicate the complex intraoperative environment of live surgery. Because this was a preclinical study performed using synthetic models, the findings should not be interpreted as evidence of improved clinical outcomes, reduced complications, or enhanced patient safety. Rather, the results demonstrate accuracy and trajectory fidelity within a controlled laboratory setting. Clinical benefit, safety, workflow efficiency, and long-term outcomes require validation in appropriately powered clinical studies. These models lack soft-tissue constraints, bleeding, and anatomical variability, which can influence tactile feedback, visualization, and real-time decision-making. Also, while the degenerative spine model used here approximates clinical pathology, it remains a simplified representation that may not fully capture the nuances of severe deformity, osteoporosis, or post-traumatic anatomy. In addition, only four surgeons participated, limiting generalizability across diverse practice settings and skill levels. Although screw-level models accounted for clustering within surgeon, the small number of participating surgeons limited the precision of surgeon-level estimates and reduced the ability to draw definitive conclusions regarding the effect of surgeon experience. Therefore, experience-stratified findings should be interpreted as exploratory and hypothesis-generating. Furthermore, this study evaluated a single robotic-assisted platform, the Mako Spine system, and the findings should not be interpreted as evidence of superiority over other robotic spine platforms. Generalizability to other robotic systems, workflows, and clinical environments therefore remains limited, and future comparative studies across multiple robotic platforms are warranted. Procedural timing and workflow efficiency were not analyzed as primary outcomes because timing measurements in the preclinical laboratory setting were not sufficiently standardized to support meaningful comparison. Future clinical studies should evaluate operative time, time per screw, radiation exposure, workflow efficiency, and learning curve in real-world surgical environments. Apart from this preclinical study, future clinical studies are warranted to validate these findings in live surgical contexts, assess the long-term outcomes of pedicle screws placed with Mako Spine assistance, and evaluate the learning curve associated with robotic adoption across different levels of surgical training.

## Conclusion

This study demonstrates that the Mako Spine system can achieve reliable, precise pedicle screw placement in a demanding preclinical model of degenerative anatomy. While the open manual technique benefits from direct visualization that generally makes accurate placement easier, the robotic platform not only produced higher rates of optimal placement and better trajectory fidelity, but did so through a minimally invasive approach. Given that MIS techniques are typically more difficult and less accurate when performed manually, these results highlight the ability of robotics to preserve the advantages of smaller exposures while maintaining precision. Taken together, the findings suggest that the Mako Spine system can reduce variability, support surgeon training, and help standardize outcomes across experience levels. Further clinical studies are needed to validate these results in live operative settings and assess long-term outcomes.

## Data Availability

The datasets generated during and/or analyzed during the current study are not publicly available, but are available from the corresponding author on reasonable request.
